# Stigma creating stigma: a vicious circle

**DOI:** 10.1192/pb.bp.114.048124

**Published:** 2014-08

**Authors:** Sokratis Dinos

**Affiliations:** 1 BPP University, London

## Abstract

Despite anti-stigma campaigns in the UK in recent years, the experiences of people with mental health problems indicate that stigma is still a major problem. The stigma of being a member of a socially excluded group, based on socioeconomic, personal or cultural/ethnic characteristics, should be considered alongside the stigma of mental illness. Membership of a stigmatised group (not based on mental illness) is often itself a risk factor for developing mental health problems. This article discusses the experiences of people from Black and minority ethnic and lesbian, gay and bisexual groups to explore how stigma can create more stigma.

## Background

The past decade has seen much activity aimed at transforming the experiences of people affected by mental health problems: specifically, trying to reduce the associated stigma. Publication of *New Horizons* set out a national vision for psychological health in England for 2010 and beyond. One of the aims was to ‘improve the mental health and well-being of the population’ by promoting equality and reducing inequality.^1^ In an attempt to achieve this goal, a number of anti-stigma government-funded programmes have been launched over the past few years (Box 1).

**Box 1** Recent and current anti-stigma and discrimination programmes in the UK and legislation

*Time to Change*: is an £18m charity-sector-led antidiscrimination and well-being campaign. As well as an advertising campaign, its work includes: a mass participation exercise, 28 community-based physical activity projects, a legal unit to pursue test cases of discrimination. Objectives over 4 years include 5% reduction in discrimination for people with mental health problems and 5% improvement in public attitudes.

*Shift*: was a Department of Health funded programme to tackle the stigma and discrimination associated with mental health issues in England. The programme was launched in 2004 by the National Institute for Mental Health in England and ran until 2011). Shift’s work complemented that of *Time to Change* by focusing on employers and the media.

*The United Nations Convention on the Rights of Persons with Disabilities*: this was ratified by the UK on 8 June 2009 and its Optional Protocol on 7 August 2009. Among its principles the convention aims to promote non-discrimination, full participation and inclusion in society, respect for difference and diversity and equality.

However, some of the latest empirical evidence of the experiences of people with mental health problems shows some strikingly similar results to evidence gathered 10 years ago.^2,3^ Similarly, researchers in the Stigma Shout survey^4^ found that nine out of ten service users reported that stigma had a negative impact on their lives and two-thirds of them cited that fear of discrimination deterred them from doing things they wanted to do.

The aims of governmental mental health strategies and the numerous anti-stigma programmes to remove stigma barriers were clear and simple. The problem is that the message may not be reaching those it is intending to reach (i.e. the general public) and/or help (i.e. stigmatised individuals). For example, a recent longitudinal survey looked at the impact of the ‘Time To Change’ campaign in changing public knowledge and behaviour^5^ and results showed no statistically significant effects in any of the indicators measured (i.e. intended behaviour, public knowledge and reported behaviour).

Attitudes and behaviours towards stigma or what constitutes stigma may not be as straightforward as anti-stigma programmes conceptualise them. Delving deeper into the background of people with mental health problems reveals that within the stigma of mental illness lies another stigma, which may be as or even more pervasive than that of mental illness; the stigma of membership in a socially excluded group based on some socioeconomic, personal or cultural/ethnic characteristics. Membership of a stigmatised group (not based on mental illness) is often itself a risk factor for developing mental health problems. As Box 2 shows, there are many groups of people who are at risk of developing mental health problems,^6^ most of which are also at greater risk of stigmatisation because of their group membership. There is evidence that membership of a socially excluded group is associated with diminished economic opportunities, poorer interpersonal relationships and other life opportunities,^7,8^ unemployment and income loss,^9^ constricted social support networks and poorer interpersonal relationships,^7^ delayed help-seeking and reduced psychiatric medication,^10^ diminished quality of life^11^ and other life opportunities,^8,12^ depressive symptoms and demoralisation,^13^ and negative constructions of identity including low self-esteem.^2,14,15^ It can be argued that stigma can create more stigma by placing someone at higher risk of developing mental health problems.

## Black and minority ethnic and lesbian, gay and bisexual groups

Looking at the example of people from Black and minority ethnic (BME) and lesbian, gay and bisexual (LGB) groups, it can be seen that both share some similarities in terms of being socially excluded based on ethnicity and/or sexuality and in having a significantly higher prevalence of mental health problems than the general population (Box 3). Both the results and the similarities between the two groups are clear. Both BME and LGB populations have a significantly higher prevalence of a number of conditions, including common mental disorders, post-traumatic stress disorder, psychosis, attempted suicide and drug dependence.

Most research points towards the higher prevalence of mental health problems among these groups being directly related to discrimination and social exclusion. Numerable surveys and systematic reviews have shown that the experience of discrimination or fear of being discriminated against can have deleterious effects on mental health and well-being (Box 4).

In addition to campaigns trying to reduce the stigma of mental illness, a number of campaigns have also been launched aiming to tackle stigma and discrimination associated with ethnicity/race and sexuality. However, similarly to the evidence on the less-than-successful outcomes of mental health programmes, research suggests BME and LGB groups still experience a significant amount of discrimination in everyday life. Such prejudice has an impact on well-being and the ability to lead a fulfilling life. For example, surveys conducted by Stonewall^27,28^ have found negative or mixed public responses towards BME and LGB groups. A total of 64% of the British population responded that they feel ‘less positive’ about at least one ethnic group.^27^ The same survey reported the general public believes that minority groups in the UK are receiving preferential treatment compared with the White majority. Although 66% of the general public believes that there is not enough acceptance of LGB people, one in five LGB people reported experiences of homophobic hate crime in the 3 years preceding the survey.^27,28^

**Box 2** Groups at risk of developing mental health problems

Children and young people

- Children with parents who have mental health or substance misuse problems
- Personal abuse or witnessing parents’ domestic violence
- Looked after children
- Excluded from school
- Teen parents
- Young offenders
- Young lesbian, gay bisexual and transgender people
- Young Black and minority ethnic groups
- Families living in socioeconomic disadvantage

Adults

- Black and minority ethnic groups
- Homeless people
- Adults with a history of violence or abuse
- Adults who misuse alcohol or substances
- Offenders and ex-offenders
- Lesbian, gay, bisexual and transgender adults
- Travellers, asylum seekers and refugees
- A history of being looked after/adopted
- People with intellectual disabilities
- Isolated older people

**Box 3** Prevalence of mental health problems in individuals who are Black and minority ethnic and lesbian, gay and bisexual

Black and minority ethnic

- Three-fold increase of people with psychosis compared with White British people.^16^ The risk in the Black Caribbean group is nearly seven-fold higher^17^
- Two-fold increase of common mental disorders in South Asian women^18^
- Two- to three-fold increase of post-traumatic stress disorder in Black men^17^
- Three-fold increase of drug dependence in Black men^18^
- On average, three-fold increase of suicide^19^

Lesbian, gay and bisexual

- Two-fold increase of suicide attempts^20^
- Over four-fold increase of lifetime prevalence of suicide attempt in gay and bisexual men^20^
- One-and-a-half-fold increase of common mental disorders and alcohol and substance misuse^20^
- 40% of lesbian or bisexual women met criteria for mental disorder and over 30% had attempted suicide^21^

**Box 4** The relationship between discrimination and mental health in Black and minority ethnic and lesbian, gay and bisexual groups

Black and minority ethnic groups

- Experience of some form of physical racial attack was associated with a prevalence of depression almost three times and a prevalence of psychosis almost five times that of people reporting no harassment^22^
- Experience of racially motivated verbal abuse or physical assault was associated with between a two- and three-fold increase in the risk of common mental disorders and psychosis^23^
- Believing the majority of British employers to be discriminatory was associated with around a two-fold increase in risk of common mental disorders^24^

Lesbian, gay and bisexual groups

• Two in three (65%) lesbian and gay pupils report homophobic bullying, including physical violence (41%) and death threats (17%)^20^
• Seven in ten gay pupils feel that homophobic bullying has affected their schoolwork and half of those who have experienced homophobic bullying have skipped school because of it^24^
• Experiences of discrimination were significantly and positively associated with depressive symptoms and anxiety symptoms^25,26^
• A significant correlation was found between experience of discrimination and having a mental disorder or attempting suicide in lesbian and bisexual women^21^

## Where next?

It seems clear there is a significant gap in the perceptions of the general public and members of stigmatised groups. The former believe such groups receive preferential treatment, whereas the latter believe they are being discriminated against and are caught in a vicious circle of a double stigma. Such beliefs of discrimination are further evidenced by the significantly higher prevalence of mental health problems among these groups. The higher prevalence of mental health problems necessitates a change of practice that will enhance accessibility and inclusion, including appropriate training provided to mental health professionals. For example, cultural and sexuality competency training regarding BME^29^ and LGB^30^ groups respectively and knowledge of needs, beliefs and lifestyles. Similarly, campaigns to educate the public do not always capture the complexity of stigma and mainly target different stigmas in isolation. From a public health point of view, further evidence about the complexity of the issue is urgently needed before any specific recommendations can be made that will feed into future anti-stigma campaigns.
